# An open-source automated PEG precipitation assay to measure the relative solubility of proteins with low material requirement

**DOI:** 10.1038/s41598-021-01126-4

**Published:** 2021-11-09

**Authors:** Marc Oeller, Pietro Sormanni, Michele Vendruscolo

**Affiliations:** Department of Chemistry, Centre for Misfolding Diseases, University of Cambridge, Cambridge, UK

**Keywords:** Analytical biochemistry, Biophysical methods

## Abstract

The solubility of proteins correlates with a variety of their properties, including function, production yield, pharmacokinetics, and formulation at high concentrations. High solubility is therefore a key requirement for the development of protein-based reagents for applications in life sciences, biotechnology, diagnostics, and therapeutics. Accurate solubility measurements, however, remain challenging and resource intensive, which limits their throughput and hence their applicability at the early stages of development pipelines, when long-lists of candidates are typically available in minute amounts. Here, we present an automated method based on the titration of a crowding agent (polyethylene glycol, PEG) to quantitatively assess relative solubility of proteins using about 200 µg of purified material. Our results demonstrate that this method is accurate and economical in material requirement and costs of reagents, which makes it suitable for high-throughput screening. This approach is freely-shared and based on a low cost, open-source liquid-handling robot. We anticipate that this method will facilitate the assessment of the developability of proteins and make it substantially more accessible.

## Introduction

Over the past decades, protein-based biologics have become a key class of therapeutics^1^. These biologics offer a range of favourable characteristics, such as high specificity and low immunogenicity, which makes them very suitable for drug discovery purposes^2^. However, proteins and antibodies destined to research, diagnostic, biotechnology, and therapeutic applications are required to endure a wide range of stresses related to manufacturing, development, shipping, storage, and administration, which they did not evolve to withstand, as these stresses are not present in vivo^3^. In particular, sub-cutaneous delivery, which is one of the most convenient ways to administer protein drugs, requires biologics to be formulated at high concentrations, so that the needed dosage can be achieved with the small injection volumes suitable for this administration route^4^. Owing to these stringent requirements, which demand protein drugs to be formulated at very high concentrations and to remain soluble and active for the shelf life of the product, solubility is a key biophysical property underpinning the developability potential^5–7^, which is defined as the likelihood of a drug candidate with suitable functionality to be developed into a manufacturable, stable, safe, and effective drug that can be formulated to high concentrations while retaining a long shelf life.

The solubility of complex macromolecules, including monoclonal antibodies and other biologics, cannot be readily defined in absolute terms, which makes quantitative assessments highly problematic^8^. The thermodynamic solubility of a substance is an equilibrium property defined as the value of the concentration—referred to as critical concentration—at which the soluble and insoluble states both present for an indefinite amount of time. While this definition is rigorous, it only applies directly to substances that, depending on the concentration, populate only two relevant states, a soluble state (the liquid phase) and an insoluble state (the solid phase)^9,10^. However, with increasing concentrations, most proteins populate a variety of metastable intermediate states, including dimers, oligomers, large aggregates and precipitates, and occasionally amyloid fibrils. Given this heterogeneity, the boundary between the soluble and the insoluble states is operationally dependent on the method used to separate the two phases, for example on the centrifugation speed or the filter size. This aspect, and the fact that some self-association pathways may lead to irreversible aggregation on the timescales relevant for therapeutic formulations, complicate the definition of protein solubility as an absolute quantity, which poses important limitations to our ability to measure solubility as an absolute value^10,11^. Despite this problem, it is possible to carry out measurements of relative solubility, which is what we do in this work. For example, it is possible to measure differences among different protein variants, or among different formulations of the same protein, in their propensity to self-associate, precipitate, or populate aggregated states, which are commonly used ways to estimate the solubility^9,11,12^.

A commonly used method to measure relative solubility of proteins is based on the use of polyethylene glycol (PEG), which employs PEG as a crowding agent to induce the precipitation of the protein under scrutiny^13,14^. It has been shown that PEG is capable of precipitating proteins out of solution without causing denaturation^13,15,16^, at least in most cases^17,18^. Many laboratories routinely use this assay, confirming its applicability to a wide range of proteins and solubility ranges^19–22^.

The mechanism by which PEG induces protein precipitation is based on excluded volume effects^15,16,23,24^, whereby the addition of polymers leads to an attractive force of entropic nature between proteins^23–25^. The association between proteins depends on the balance between the enthalpy gain due to their interaction and the entropy loss due to the formation of a bound state. More specifically, by reducing the volume available to the unbound proteins, a crowding agent reduces the entropy loss upon the binding of proteins, and conversely, this binding increases the volume accessible to the crowding agent increasing its entropy. The effective protein concentration can thus be increased by increasing the concentration of PEG, until the critical concentration is exceeded, and precipitation occurs.

In recent years, several groups introduced improvements on the PEG assay by reducing protein demand and increasing reliability by automation. The reliability of this assay has been shown by comparing its results with ultracentrifugation^22^ and ultrafiltration^19^, demonstrating that it represents an excellent alternative to more resource-intensive approaches to assess relative protein solubility. This method offers distinctive advantages to investigate protein solubility, including high accuracy, relatively low material consumption, and the lack of requirements for any highly specialised and expensive piece of equipment or material. With the possibility of being automated using a pipetting robot, the employment of PEG precipitation assays becomes even more convenient. However, automated liquid-handling robots have traditionally been very expensive and required highly trained personnel for their operation, which prevented their widespread dissemination especially in smaller academic labs. Furthermore, although the material requirements for PEG precipitation assays are greatly reduced compared to other approaches that afford similar accuracies (dynamic light scattering, ultracentrifugation, etc.), milligrams of material are typically required.

Recent advances in open hardware and increasing efforts to democratize lab robotics have resulted in a sharp decrease in the price-tag of liquid handling robots, while maintaining high accuracy and fast run times^26^. Similarly, open-source software to programme these robots, and the ability to share protocols across labs are substantially increasing their ease of use^27,28^. Here, we exploit these advances to introduce a PEG precipitation assay that runs in a fully automated way, and with low material consumption. Automation delivers a range of benefits, from improved reproducibility and accuracy, to scalability and walk-away time. By using a cost-effective open-source pipetting robot we remove one of the major hurdles to implement these approaches. We further make our protocols publicly available and open source, to ensure widespread dissemination and easiness of implementation.

## Results

### PEG precipitation assay for protein solubility measurements

**Figure 1:**
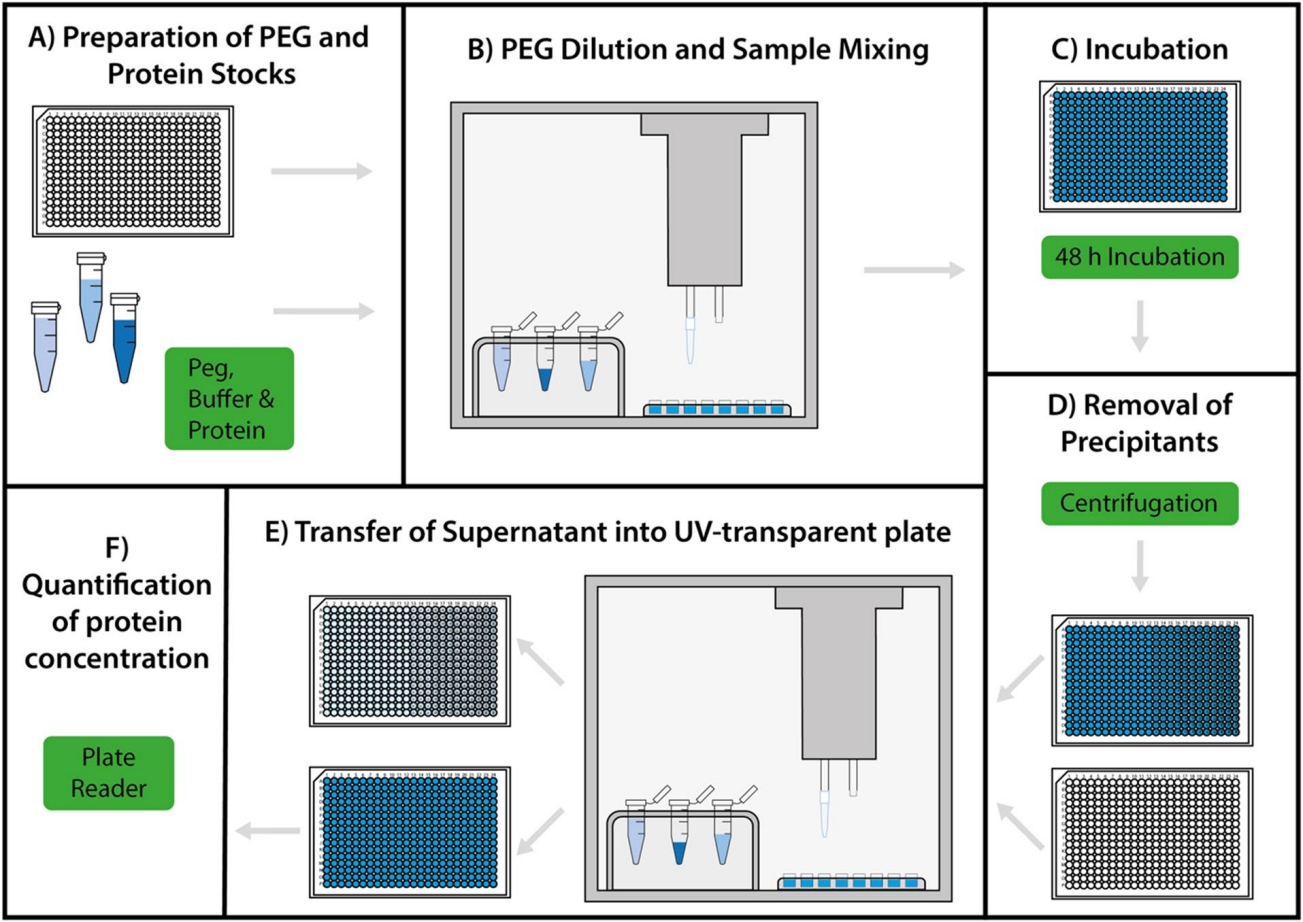
Outline of the procedure described in this article for the measurement of protein relative solubility using an automated PEG assay. **(A)** All necessary material, including buffer, PEG solutions, proteins and an empty plate are prepared. **(B)** The robot titrates PEG and performs sample mixing for a final well volume of 10 µL. (**C)** After sealing, the plate is incubated for 48 h at 4 °C. **(D)** The precipitates are separated from the solution by centrifugation. **(E)** The supernatant is transferred into a freshly prepared UV-transparent plate. **(F)** The protein concentration in the supernatant (i.e. the soluble concentration) is estimated with absorbance measurements carried out with a plate reader.

The procedure for the automated PEG precipitation assay that we developed for measuring protein solubility comprises 6 steps (Fig. 1): (A) preparation of PEG and protein stocks, (B) PEG dilution and sample mixing, (C) incubation, (D) centrifugation to spin-down precipitates, (E) transfer of the supernatant to a UV-transparent plate, and (F) quantification of supernatant protein concentration by absorbance measurements. The most time-consuming steps (B) and (E) have been fully automated by writing customised protocols for an open-source pipetting robot (see “Methods” section).

We run our assay at a final protein concentration of 1 mg/mL, which is standard in the literature^12,19,29^, and with a final volume of 10 µL per well in 384-well low-volume microplates. If needed, all these quantities can easily be adjusted to user-specific needs with minor edits of the robot protocols. Our procedure does not require particularly long or complicated preparation steps. It is, however, important to ensure that the PEG is thoroughly dissolved in a stock solution made of the same buffer later used in the assay for the PEG titration, and that the pH of this stock solution is re-adjusted following addition of PEG (Fig. 1A). Usually for these kinds of precipitation assay, the cumbersome, time-consuming, and often inaccurate step is the titration of highly concentrated, viscous PEG solutions into a low-volume multi-well plate. By using a pipetting robot, we save manual labour, increase accuracy and reproducibility, and reduce required sample volume and execution time (Fig. 1B). Solutions that contain more than 20% PEG are very viscous, which makes it difficult to pipette them accurately. By maximising the accuracy of automated pipetting of viscous solution, we are able to use only 10 µL of total sample volume per well.

**Figure 2:**
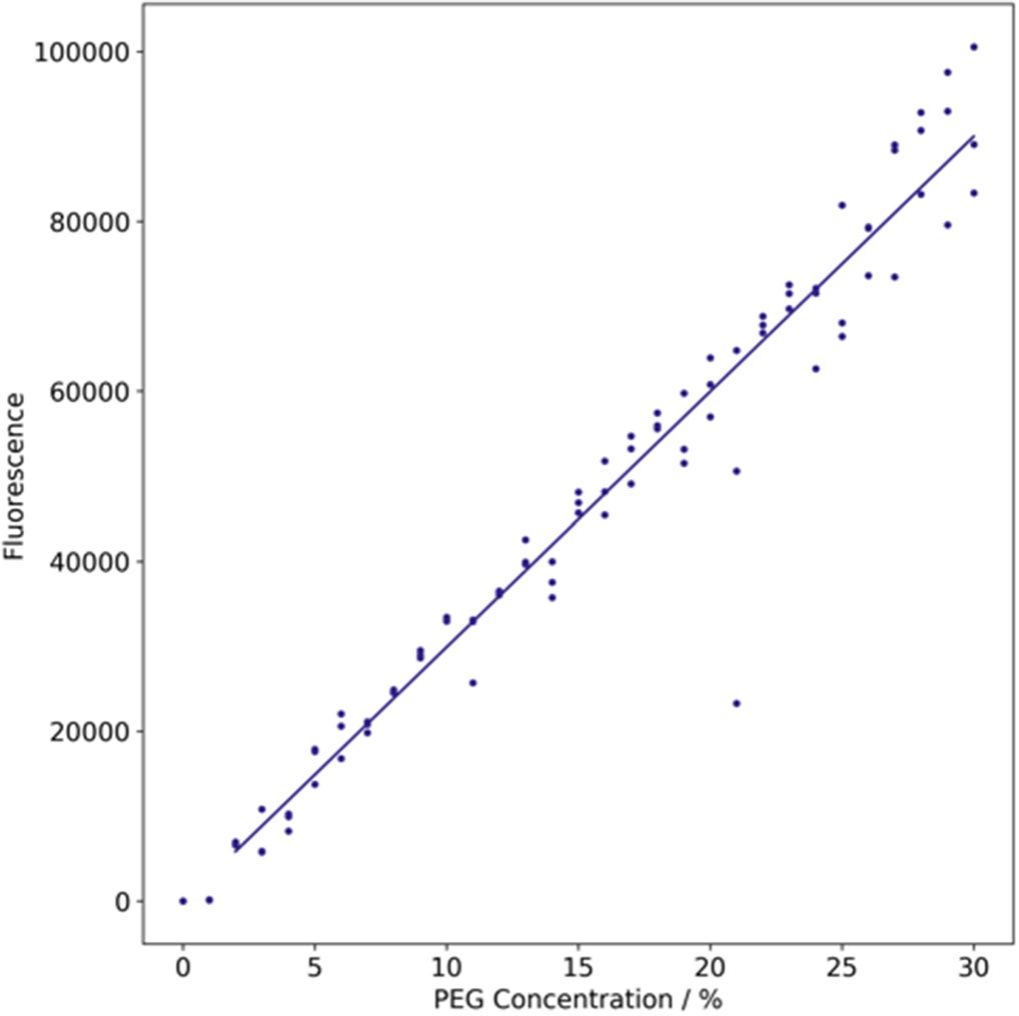
The robot pipetting is accurate despite the high viscosity of PEG-containing solutions. The automated PEG assay was carried out by titrating a PEG stock containing free AlexaFluor488 fluorophore. We validated the accuracy of the robot in transferring solutions by assessing the linear relationship between the AlexaFluor488 fluorescence (y-axis) and the expected PEG concentration (x-axis). The median error which represents the shift from the expected PEG concentration is about 0.8%.

To demonstrate that despite the high viscosity of concentrated PEG solutions the pipetting is accurate, we ran the assay by adding AlexaFluor488 to the stock of 50% PEG. We then ran a standard PEG precipitation protocol, but instead of adding protein, buffer containing PEG and free AlexaFluor488 was titrated. With this setup, we expect the fluorescence intensity of AlexaFluor488 to linearly increase with the PEG concentration (Fig. 2). The measured AlexaFluor488 fluorescence as a function of the expected PEG concentration (expressed as a weight/volume percent) reveals that even volumes as low as 0.33 µL are transferred rather accurately, as the median error of the final PEG concentration is about 0.8% (Fig. 2).

Overall, for a standard run using twelve PEG concentrations with two replicates, only 80 µL of protein at a stock concentration of 3 mg/mL are needed, corresponding to a requirement of 240 µg of purified protein per assay. This amount is calibrated for a reasonable screening-step assay, but can be further reduced by reducing the number of data points or by doing only one replicate per concentration, which can further increase throughput when screening a large number of variants. In general, 10–12 data points across a wide range of PEG concentrations is enough to determine the position of the half-point of the sigmoidal curve. If very accurate results are required, a more suitable selection of PEG concentrations around the slope can be chosen to further increase the accuracy of the fit.

Using our default implementation, which assumes a protein stock concentration of 3 mg/mL, a concentration range of 0–33% PEG (weight/volume) can be achieved, which is comparable with methods developed by other groups and is suitable for most proteins^21,22^. Nonetheless, as the PEG stock solution is at 50%, using a higher stock protein concentration (> 3 mg/mL) would make it possible to readily increase this range to up to 45% PEG if needed for highly soluble proteins. By employing single-channel pipettes, it takes the robot about 70 min to finish the preparation of a standard run with 12 data points including two replicates and one control (i.e. blank) per concentration. The time could be further reduced by employing a multi-channel pipette, by having only one well as a blank given that PEG does not absorb at 280 nm (albeit at very high concentrations it can cause some scattering in this range), or by speeding up the movement of the pipette or the aspiration/dispensing speed. We found, however, that to ensure high accuracy, slower speeds are preferred.

After sample preparation, the plate is sealed and incubated for 48 h at 4 °C to fully equilibrate (Fig. 1C)^11,12^. Then, the plate is centrifuged at maximum speed (2000 g) for 2 h to pellet down any precipitated protein material (Fig. 1D). Prior to centrifugation, one may also carry out a turbidity measurement provided the sample preparation was done in a clear-bottom plate. While simpler in its implementation, we find that turbidity provides a less accurate readout than the quantification of the soluble-fraction concentration (see “Turbidity vs. supernatant absorbance concentration measurements” section), especially for proteins of low molecular weight.

Following the centrifugation, the supernatant is transferred using the robot into a UV-transparent plate prefilled with the same buffer used before (Fig. 1E). Specifically, to ensure that the supernatant is transferred without disturbing the pellet, the protocol takes 3 µL of supernatant from each well twice (i.e. 6 µL out of a total of 10 µL, which may have decreased down to 8 µL because of evaporation). If only 3 µL of liquid were used, the path length would be inconsistent, as this amount is not enough to cover the bottom of the well. Hence, concentration readouts would be affected by large errors, which makes it necessary to dilute the sample in a larger volume. While there are specialised ‘drop-sense’ readers that can accurately measure concentrations in microdrops, our aim is to make this protocol as broadly applicable as possible using standard microplate readers and pipetting robots.

The transfer of supernatant after centrifugation is time-critical, and needs to be carried out as soon as possible to prevent the resuspension of any precipitate. Automation increases speed and well-to-well consistency. From each well of the sample plate, two transfer steps can be carried out to double the number of technical replicates in the dilution plate. Finally, the soluble concentration of the protein present in the supernatant can be determined by measuring the absorbance with a plate reader from a total of 4 wells per concentration and 2 per blank (Fig. 1F).

### Data analysis

**Figure 3:**
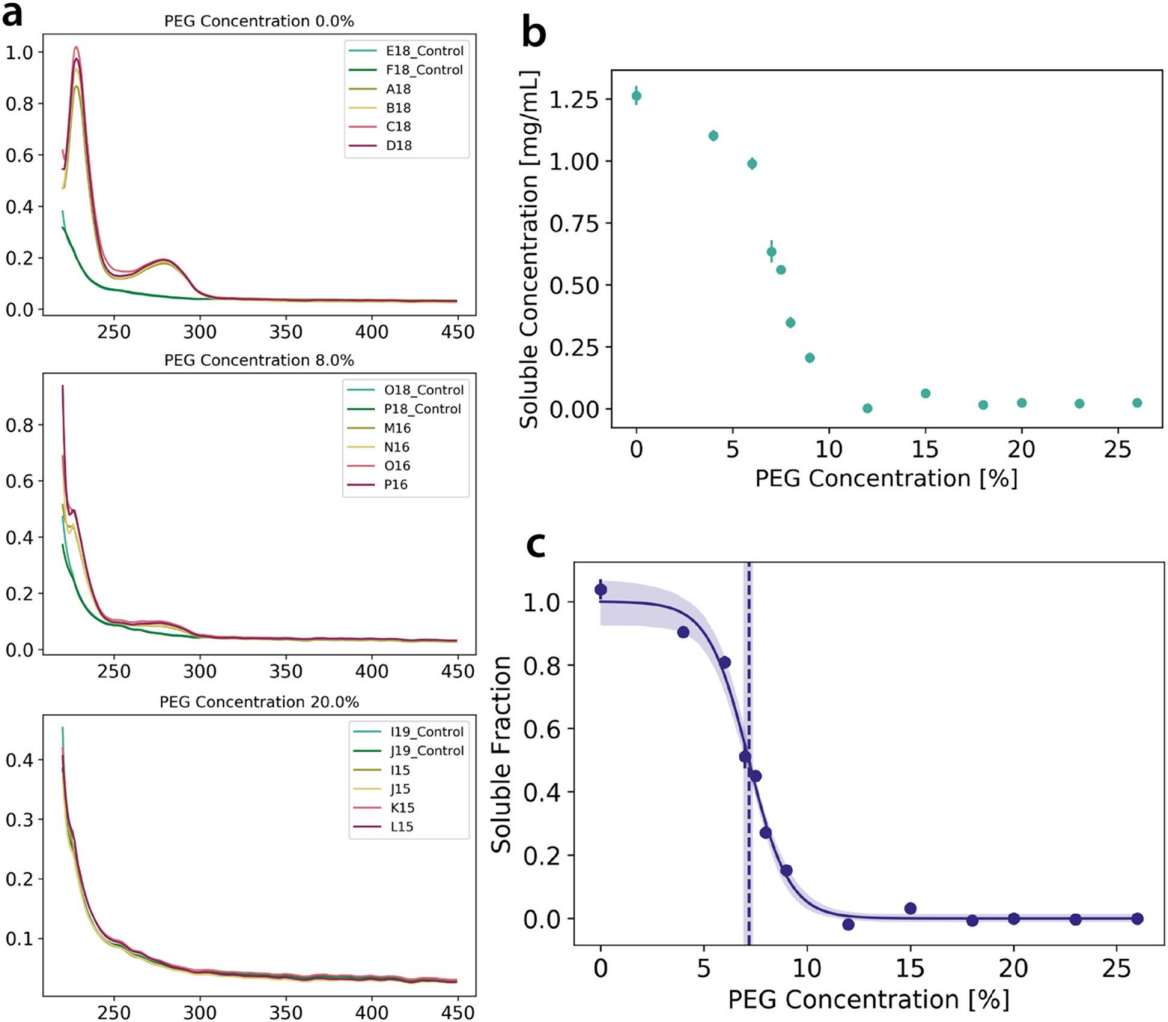
Step-by-step data acquisition and fitting.** (a)** In a pre-analysis step, possible outliers in the measurements are identified from the raw data, as described in the Data Analysis section. The absorbance profiles are then analysed with their suitable blanks to obtain the protein concentration in the supernatant. (**b)** For each PEG concentration, the soluble concentration of the protein is calculated from the absorbance at 280 nm and plotted. (**c)** Following a normalisation step, a sigmoid function is fitted through the soluble fraction to acquire the PEG_1/2_ value as a proxy for relative solubility. A 95% confidence interval analysis is carried out here to estimate the error on the PEG_1/2_ and the quality of the fit.

The analysis of the PEG-precipitation data is carried out through a series of steps (Fig. 3). After pre-processing, the raw data is inspected for outliers. Measurements are identified as outliers if:

1. the absorbance value at 310 nm is outside the expected range. The expected range is defined as the mean ± 4 × the standard deviation at 310 nm (i.e. 4-σ away)
2. the absorbance values averaged in the range from 350 to 400 nm is outside the expected range. The expected range is defined as the mean ± 7 × the standard deviation (i.e. 7-σ away).

Reference means and standard deviations are obtained by compiling raw data from eight different experiments with eight different proteins. Both criteria aim at removing measurements that are affected by scattering from air bubbles or other large inconsistencies not attributable to actual protein precipitation. The expected range was defined by closely analysing these eight different runs to ensure that the inclusion criteria are stringent enough to exclude all measurements affected by scattering while at the same time not excluding valid measurements.

The protein concentration in each well is calculated from the absorbance at 280 nm and visualised as a plot of the soluble protein concentration against PEG concentration (Fig. 3b). From this, a sigmoidal curve is fitted and normalised to determine PEG_1/2_ and its 95% confidence interval (Fig. 3c). Furthermore, additional parameters such as the slope and the error of the fit can be collected for more in-depth analysis.

### Application to the tool antibody HzATNP and to bovine serum albumin (BSA)

**Figure 4:**
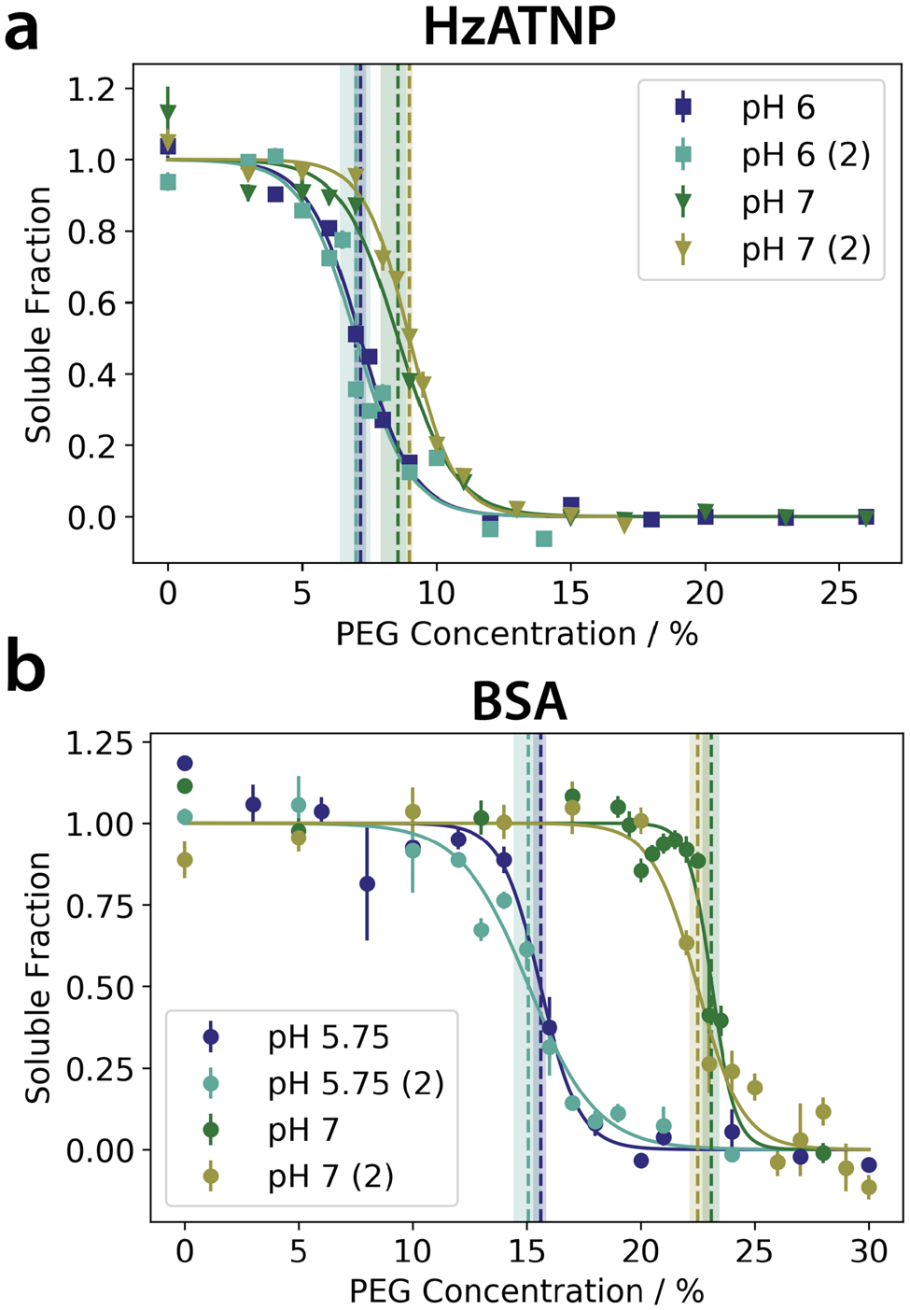
Accuracy and reproducibility of the automated PEG assay to measure the relative solubility of proteins at different pH values. The results of four independent measurements of the antibody HzATNP **(a)** and of BSA **(b)** at two different pH values highlight the accuracy and reproducibility of this assay. Vertical lines represent PEG_1/2_ values including their 95% confidence intervals. The legend described the buffer pH and (2) denotes a second independent experiment carried out on a different day from a fresh sample preparation.

In order to validate our approach, we first applied it to measure the relative solubility of the monoclonal antibody HzATNP (wild-type monoclonal antibody (mAb) in Ref.^11^) and bovine serum albumin (BSA) at different pH values (Fig. 4). The 95% confidence interval analysis on the fitted PEG_1/2_ indicates that the assay is accurate enough to distinguish the solubility of the antibody at the pH values investigated here. Moreover, it shows that the results are reproducible, as independent runs done in different days for each pH overlay very closely, and yielded PEG_1/2_ values with overlapping confidence intervals.

### Turbidity versus supernatant absorbance concentration measurements

**Figure 5:**
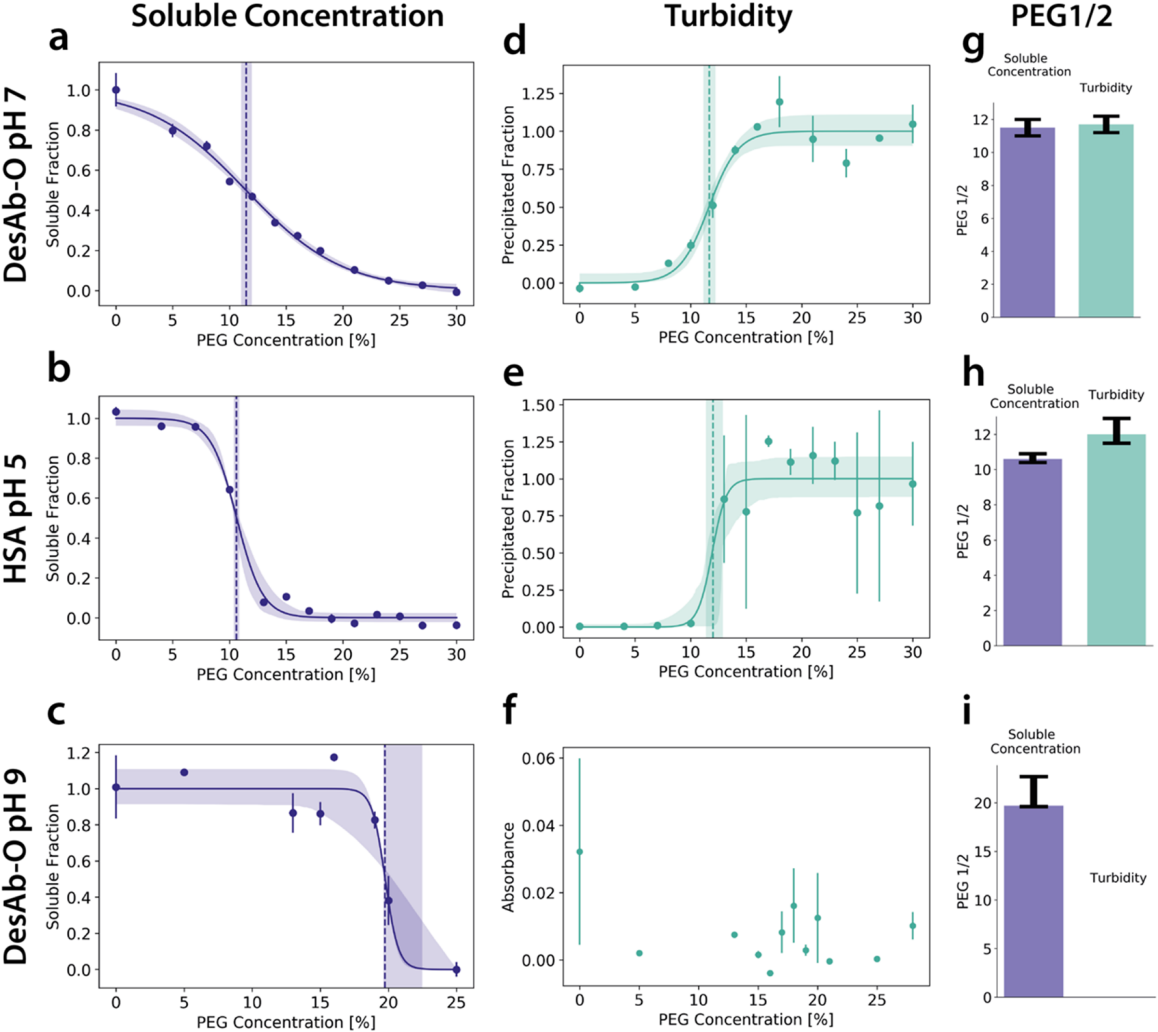
To quantify soluble concentrations, supernatant absorbance measurements are more consistent than turbidity measurements.** (a–c)** Absorbance measurements of soluble fractions after transfer of the supernatant**. (d–f)** Turbidity measurements of the same run taken before centrifugation. **(g–i)** Bar plots comparing the PEG_1/2_ values for absorbance of the soluble concentration and turbidity. (**a, d, g)** PEG_1/2_ values for both approaches agree for DesAb-O at pH 7. (**c, e, h)** For HSA at pH 5, PEG_1/2_ values are affected by small errors (**b, h)**, while the turbidity shows a later onset of the precipitation and has larger error bars across technical replicates. **(e, h)**. **(c, f, i)** While absorbance measurements show a clear drop at around 20% PEG for DesAb-O at pH 9, indicating that the protein is precipitating, the turbidity measurement data do not reliably detect changes because of the high level of noise.

As protein precipitation increases the turbidity of the solution, it is in principle possible to stop our assay after step C (Fig. 1) and directly measure the turbidity of the samples, skipping in this way the time-consuming processes of centrifugation and supernatant transfer. Indeed, this approach has been adopted several times in the literature, as it offers a fast route to determining PEG_1/2_ and hence the relative solubility^22^. In our tests, measurements of turbidity and of corresponding supernatant concentrations by absorbance yielded similar PEG_1/2_ values, at least for some proteins and buffer conditions (Fig. 5a–c). However, in other cases, we observed that although a sigmoidal curve was clearly observed in both types of measurement, turbidity data were characterised by much larger error bars and yielded slightly different PEG_1/2_ estimates (Fig. 5d–f). In addition, in a few cases where we measured the solubility of highly soluble, low molecular weight species, we saw a drop in the absorbance measurement but could not identify a PEG_1/2_ value for the turbidity measurement (Fig. 5g–i). Overall, absorbance measurements of supernatant concentrations yielded more consistent results in our experiments than turbidity measurements.

## Discussion and conclusions

In this study we have presented an open-source automated PEG precipitation assay to measure accurately the relative solubility of proteins with low material requirement. We have validated this assay by applying it to a range of different proteins and buffer conditions.

Our goal is to make this assay as broadly applicable as possible, in particular at the earlier stages of development. Therefore, we have focussed our efforts on reducing material requirements and on making the method automated and accessible, to ensure widespread dissemination and easiness of implementation.

Previous studies showed that the PEG precipitation method works for both low and high solubility compounds^20,30,31^. Our work confirms that this assay works for proteins with a range of varying solubilities and sizes. Ranging from nanobodies with a molecular weight of 14 kDa to BSA with 66 kDa up to full-length antibodies weighing about 150 kDa.

It is important to note that this assay measures relative solubility between either similar proteins or the same protein at different conditions and is not intended for comparing proteins that differ substantially in sequence and structure^22^. One reason for this limitation is that although PEG is mostly inert some proteins can interact with PEG and their behaviour in solution might be slightly altered^17,18,32^. This issue however does not affect measurements of relative solubility, as the ranking among protein variants originating from the same wild-type, or of different formulations of the same protein should be conserved. Another point worth highlighting is the possibility of evaporation, which is greatly reduced by sealing the plate tightly and incubating it at 4 °C. However, some evaporation is still observed, and the level of evaporation might in principle slightly change for different proteins and be affected by the PEG concentration. This is one of the reasons we kept the incubation time constant across all experiments, although 24 h might be sufficient for most proteins, complex proteins benefit from longer incubation times. As long as the incubation time of 48 h is kept constant, the level of evaporation should remain the same across different experiments and should therefore not affect rankings of relative solubility obtained with this assay.

As a solubility proxy to assess the relative solubility, we chose PEG_1/2_ which represents the PEG concentration at which 50% of the protein is still soluble. Other groups have made use of the same approach^19,21,24^, while others used the onset of the sigmoidal curve^22^, which can also readily be obtained from our analysis pipeline. Taken together, these reports indicate that the measurement of PEG_1/2_ is suitable for high-throughput solubility screening of protein variants^19^. When it comes to more detailed analyses, however, it is important to note this approach is not well suited to measure the absolute solubility. Extrapolating the slope of the curve at PEG_1/2_ to 0% PEG on a log-scale offers a way to estimate the apparent absolute solubility^19^, but it is not suitable for high-throughput screening, as it is extremely sensitive to minor inaccuracies (Fig. S1). Nevertheless, it can prove useful in more accurate measurements with high material requirements^19^.

In our hands, absorbance measurements of soluble concentration proved more reliable than turbidity measurements, albeit the latter are easier to obtain as they don’t require to separate out the precipitate^19–22,24^. Overall, we recommend using supernatant absorbance as it seems to yield more consistent results.

Our assay provides two major advantages over previously published assays of this type. First, the material requirements are considerably reduced. Compared to previous methods, our method saves 60–90% of purified protein while retaining similar accuracy^21,22^. This was achieved by reducing the sample volume down to 10 µL per well, which is not possible with manual pipetting due to the high viscosity of PEG. However, using a pipetting robot and putting substantial efforts into finding appropriate tailored pipetting settings we were able to accurately transfer very low amounts of highly concentrated PEG.

The material requirement could be further reduced by using smaller final protein concentrations. We used 1 mg/mL as this is the standard for solubility assays. This concentration, however, is highly protein dependent and is affected by the protein’s solubility and absorbance. Large proteins that absorb readily in the UV region, such as BSA are likely to work with this assay at lower concentrations as well. However, small proteins that do not readily absorb in the UV region need higher concentrations to overcome low signal to noise ratios from measuring absorbance with a plate reader. Other concentration determining methods might be employed for these proteins. Here, we kept the concentration constant across all experiments to avoid deviating from the main procedure.

Low material requirement also contributes to increased throughput. Using only single channel pipettes it takes around 70 min to prepare one protein with 36 data points (two replicates and one control per PEG concentration). The transfer of the supernatant two days later takes another 45 min. Employing multichannel pipettes, this throughput can be increased up to eightfold leading to the possibility of screening around 50 proteins during a standard 8-h workday.

In addition to decreasing material requirement, the method that we have described aims at removing other accessibility hurdles as well. We implemented our approach exploiting an open-source automated pipetting robot that is more affordable than standard pipetting robots, and we provide the community with all the protocols to run the robot, and analysis scripts to replicate our results. Assuming that a laboratory that carries out developability research already owns a centrifuge with a plate rotor and a plate reader for absorbance measurement, the only cost that arises from implementing this assay is stemming from the pipetting robot. For example, the Opentrons OT-2 robot with open-source software we have employed can be set up with less than £10,000, which is several folds cheaper than other state-of-the-art liquid handling robots. This approach is aligned to the frontiers of open science and will greatly facilitate technology transfer by enabling the easy implementation of our workflow in other laboratories.

## Materials and methods

### Plates

This assay can be performed with any 96- or 384-well plate. We recommend using low-volume 384-well plates to reduce the material requirement as much as possible (Greiner, 788876). Aluminium plate sealers were most effective at preventing evaporation (Corning, 6570).

### Buffer

10 mM citrate 10 mM phosphate buffer were prepared by dissolving the appropriate mass of citric acid monohydrate (Fisher Scientific, 5949-29-1) and sodium phosphate dibasic heptahydrate (MP Biomedicals, 191441) in MiliQ water. Subsequently, the pH was adjusted to the desired value using a pH meter and stock solutions of 1 M NaOH or 1 M HCl.

### PEG preparation

PEG stocks were prepared by dissolving polyethylene glycol 6000 (Sigma Aldrich, 81,260) in the previously prepared 10 mM citrate 10 mM phosphate buffer to reach a concentration of 15%, 30% and 50% PEG (weight/volume). To dissolve PEG, heating up the solution to around 50 °C is necessary. The pH shifted during the dissolving of PEG and had to be adjusted again. Adjusting the pH of highly concentrated PEG solutions is difficult and takes a considerable amount of time. It is important to let the solution stir for at least 2 min every time acid or base is added to ensure complete mixing before measuring the pH again. Due to this complication, it is suggested to prepare the PEG solutions at least one day in advance of starting the assay. This ensures complete mixing and on the day of the assay the pH can be checked and adjusted again if necessary.

### Protein preparation

The monoclonal antibody HzATNP was provided by Novo Nordisk and prepared as described in ref. 11. DesAb-O was expressed and purified in our lab (as described in ref. 30) BSA (SIGMA, A9418) and human serum albumin (HSA, Merck Life Science UK Limited, A3782) were dissolved in buffer at pH 5.75 and further purified by gel filtration using a Superdex 200 column. HzATNP and DesAb-O were buffer exchanged into the previously described 10 mM citrate, 10 mM phosphate buffer at the pH values specified in the results section. Right before use, proteins were concentrated using 0.5 mL Amicon Ultra Filtertubes (Merck Millipore, UFC500396) to a concentration of 3 mg/mL and kept on ice. Protein concentrations were measured by their Abs280 values using NanoDrop (Thermo Scientific, NanoDrop 2000), and extinction coefficients were obtained from the sequence using the Expasy ProtParam web server.

### Protocols and analysis

All protocols used in this project are available on GitLab under https://gitlab.developers.cam.ac.uk/ch/sormanni. All of these protocols can be used directly with the Opentrons software and a description of each protocol is provided on GitLab as well.

### Absorbance measurements

Absorbance of the soluble fraction and turbidity was measured with a plate reader (BMG Clariostar). The spectrum from 220 to 700 nm was recorded at 25 °C with 100 flashes per well. Turbidity was defined as blanked absorbance at 500 nm, while protein concentration was calculated from the blanked absorbance at 280 nm further corrected from that at 340 nm.

### Jupyter notebook—Plate Reader Analysis

This notebook guides the user through the analysis of the experimental data. Details about each function are given in the notebook. Here, only a brief overview over the different steps is given. At first, the pre-processed raw data are loaded in. The input should be a csv file containing the following information for each well: PEG concentration, whether it contains protein or a control and absorbance values for all wavelengths measured. A script that pre-processes ascii and excel files from BMG plate readers is available on GitLab. It also applies a Savgol filter to smooth the data and reduce the noise. Then the user is prompted to run a script that identifies false readings that should be excluded from further analysis. Subsequently, the soluble protein concentration against PEG concentration is plotted. This is done by calculating the concentration at 280 nm while controlling for the absorbance at 340 nm. Finally, the sigmoidal fit is performed. The function being fitted is

$$ y = \frac{a - b}{{1 + e^{{s\left( {x - PEG_{1/2} } \right)}} }} + b, $$

where a and b constitute the upper and lower plateaus, s is the slope of the curve and PEG_1/2_ is the inflection point.

The fit contains several steps. After fitting an initial sigmoidal function, the data are normalised by using the upper and lower plateaus. With the normalised data 95% confidence intervals are calculated with 500 bootstrap cycles. The notebook also contains functions to analyse turbidity and intrinsic fluorescence.

### Steps outline

#### Day-1

- Buffer and peg preparation

- At least 1 day in advance to correct pH shift

#### Day 2

- Check buffer and PEG pH
- Buffer exchange protein if necessary
- Concentrate protein if necessary
- Set labware location and concentrations and volumes of buffer, PEG solutions and protein stock in PEG_Assay_Step1.py protocol
- Add all necessary reagents
- Calibrate labware
- Start PEG_Assay_Step1.py protocol
- Seal plate and incubate at 4 °C for 48 h

#### Day 3

- Centrifuge plate for 2 h at maximum speed (2272×*g*)
- Set labware location, buffer volume and add which wells are to be transferred from the assay plate into the fresh plate in PEG_Assay_Step2.py protocol
- Add all necessary reagents
- Calibrate labware
- Start the protocol to transfer dilution buffer into fresh plate while the assay plate is centrifuging. It will pause automatically once finished.
- Add centrifuged plate to robot and resume the protocol to transfer two times 3 µL of the supernatant into measurement plate.
- Measure protein concentration using a plate reader.

## Supplementary Information

Supplementary Information.

## Abbreviations

BSA: Bovine serum albumin
PEG: Polyethylene glycol
