# Supplementary material for: An open-source automated PEG precipitation assay to measure the relative solubility of proteins with low material requirement
Source: Sci Rep. 2021 Nov 9;11:21932. doi: 10.1038/s41598-021-01126-4 (PMC8578320; doi:10.1038/s41598-021-01126-4)
Supplement: Supplementary file 1 — Supplementary Information. [file 41598_2021_1126_MOESM1_ESM.pdf]

Supporting Information

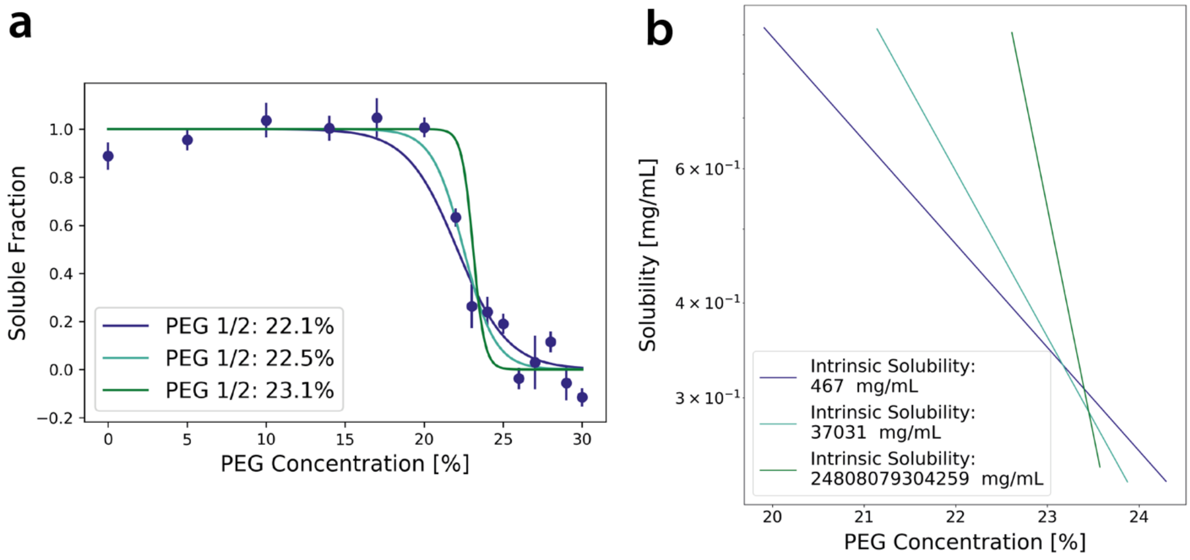

Figure S1. Small experimental or fitting errors can cause extreme errors in the calculation of the solubility. (a) Three sigmoidal fits through the same experimental data representing the 2.5% (blue), 50% (purple), and 97.5% (green) percentiles of the bootstrapped fitting parameters yielding the best $PEG_{1/2}$ and its C.I. (legend). The three fits result in very similar $PEG_{1/2}$ values, indicating a narrow C.I. (**b**) Extrapolation of the sigmoidal fits using the fitted slopes from panel A results in 11 orders of magnitude difference in the estimate of the apparent solubility (legend).
